# The Association of Neovascularization With Optical Coherence Tomography Angiography Parameters in Proliferative Diabetic Retinopathy

**DOI:** 10.7759/cureus.39633

**Published:** 2023-05-29

**Authors:** Punita K Sodhi, Ekta Shaw, Akanksha Gautam, Kavya C Rao, Archana T R, Bratati Banerjee, Anju Rastogi

**Affiliations:** 1 Ophthalmology, Guru Nanak Eye Centre & Maulana Azad Medical College, New Delhi, IND; 2 Ophthalmology, Dr. Rajendra Prasad Centre for Ophthalmic Sciences, All India Institute of Medical Sciences, New Delhi, IND; 3 Community Medicine, Maulana Azad Medical College, New Delhi, IND

**Keywords:** vascular density, superficial capillary plexus, proliferative diabetic retinopathy, optical coherence tomography angiography, neovascularization, foveal avascular zone, diabetic retinopathy, deep capillary plexus

## Abstract

Aim: We aim to find an association between neovascularization (NVn) and optical coherence tomography angiography (OCTA) parameters in proliferative diabetic retinopathy (PDR).

Methods: In a prospective study, 41 subjects including 28 (68%) males and 13 (32%) females having PDR were examined for neovascularization disc (NVD) and neovascularization elsewhere (NVE) clinically and with fundus fluorescein angiography (FFA). A total of 79 eyes were found to be involved. We examined OCTA parameters including foveal avascular zone (FAZ) size, perimeter and circularity, and vessel density (VD) in the superficial capillary plexus (SCP), deep capillary plexus (DCP), outer retina (OR), outer retinal chorio-capillaries (ORCC), chorio-capillaries (CC), and choroid (C) in these subjects.

Results: In eyes with NVD, the central foveal thickness (CFT) (p=0.83) and sub-foveal choroidal thickness (SFCT) (p=0.08) were higher, the FAZ area was significantly larger (p=0.005), and the VD was lower in all retino-choroidal layers. However, it was significantly lower in SCP foveal (p=0.005) and ORCC foveal (p=0.05) than in eyes not having NVD. For NVE, the CFT (p=0.03) and SFCT (p=0.01) were more in affected eyes. The eyes without NVE had a better circularity index (p=0.07) and the highest VD in OR slab (p=0.02) than those eyes that had NVE < ½ disc area (DA) and NVE > ½ DA. On comparing eyes without NVE, NVE < ½ DA, and NVE > ½ DA, the latest had the highest VD in SCP (p=0.59) and lowest VD in DCP (p=0.43) and OR (p=0.02). The VD in ORCC, CC, and choroid was highest in the no NVE group, followed by the NVE > ½ DA and NVE < ½ DA groups in that order. The subjects having vitreous hemorrhage (VH) and intra-retinal microvascular abnormalities (IRMA) had higher values for CFT and SFCT than eyes without these.

Conclusions: An increased CFT and SFCT are associated with the appearance of NVD, NVE, VH, and IRMA. The presence of NVD, VH, and IRMA is associated with a larger FAZ area, while that of IRMA and NVE is associated with reduced FAZ circularity. Eyes with NVD, VH, and IRMA had lesser VD in all the retino-choroidal layers. Eyes with NVE > ½ DA had the highest VD in SCP and lowest in DCP and OR; this pattern of VD foretells severer affection in NVE. IRMA was associated with a larger FAZ area, larger FAZ perimeter, and lesser circularity, indicating the presence of central ischemia.

## Introduction

Diabetes mellitus (DM) is a chronic disease with an overwhelming impact on public health and is a leading cause of blindness worldwide. Recent evidence has emerged that diabetic retinopathy (DR) affects more than 90 million people, of which 17 million have proliferative diabetic retinopathy (PDR) [1]. PDR is characterized by retinal neovessels (NV) at the disc and elsewhere, growing into the vitreous through a break of the internal limiting membrane (ILM) to cause severe visual loss (SVL) through tractional retinal detachment (TRD) and vitreous hemorrhage (VH) [2].

The diagnosis of NV is established clinically through standard and wide-field fundus fluorescein angiography (FFA), which has shortcomings such as the inability to detect microvascular changes and obscuration of details from dye leakage. Optical coherence tomography angiography (OCTA) can visualize microvascular systems at different retino-choroidal levels. In addition to showing a capillary dropout and pruning of vessels, it documents macular ischemia by delineating and measuring foveal avascular zone (FAZ) parameters and finding vessel density (VD) values [3,4]. The equipment can differentiate NV, collaterals, and intra-retinal microvascular abnormalities (IRMA) as NV is raised and is visible in low resolution. The collaterals are in the retino-choroidal plane and are seen in a higher resolution. IRMA are documented as vessel abnormalities that have not breached the ILM.

The examination of microvascular changes can tell about the perfusion status of the retina and the likelihood of developing neovascularization (NVn). During the progress of NVn in PDR, both the FAZ and VD parameters of retino-choroidal layers are expected to be adversely affected. Although there are some studies on OCTA in NVn from PDR [5-8], there needs to be more literature on the association between NVn and OCTA parameters. This study aimed to find an association between features of NVn with OCTA parameters in subjects of PDR.

This study was originally presented as a free paper at the 80th All India Ophthalmological Conference 2022, Mumbai, India, on June 2, 2022.

## Materials and methods

We conducted a prospective study on subjects aged 40-70 years with PDR as per Early Treatment Diabetic Retinopathy Study (ETDRS) Classification [9]. The study extended for a period of one and a half years from December 2019 to June 2021. The study was conducted in compliance with the tenets of the Declaration of Helsinki and was approved by the Institutional Ethics Committee vide letter number F. 1/IEC/MAMC/(70/05/2019/No. 506 dated 01/11/19). The study was performed at the Department of Ophthalmology, Guru Nanak Eye Centre and Maulana Azad Medical College, New Delhi.

We took a history of ocular symptoms such as a diminution of vision, metamorphopsia, decreased contrast sensitivity (CS), and defective color vision (CV), duration of diabetes, and other systemic diseases. Subjects with vascular abnormalities such as neovascularization disc (NVD) and neovascularization elsewhere (NVE), IRMA, and VH were included in the study. We excluded subjects with other significant ocular or systemic diseases such as glaucoma, uveitis, media opacity, optic atrophy, high refractive error, central nervous system disease or tumors, and previous DR treatment with anti-vascular endothelial growth factor (VEGF) and laser. Subjects with proliferative retinopathy from other causes such as retinal vascular occlusion, sickle cell retinopathy, ocular ischemic syndrome, or radiation retinopathy were also eliminated.

Following informed consent, a detailed ocular examination of both eyes separately was done for baseline evaluation of best corrected visual acuity (BCVA) on ETDRS Charts at 4 m under uniform illumination. Additionally, we measured intraocular pressure (IOP) with an applanation tonometer and did a retinoscopy, dilated fundus examination, and fundus photograph. We included only subjects having BCVA of at least a logarithm of the minimum angle of resolution LogMAR 1.0 (20/20, 6/60) in the study. We examined CV on the Farnsworth D-15 test and CS on Pelli Robson Charts (Haag-Streit Service, Inc., OH, USA). The spectral domain optical coherence tomography (OCT) (SD-OCT) Nidek (RS-3000, Software NAVIS-EX; Fremont, CA, USA) was used to examine intra-retinal edema, exudates, cystoid macular edema (CME), central foveal thickness (CFT), sub-foveal choroidal thickness (SFCT), and the status of the posterior hyaloid. The CFT was defined as the average retinal thickness within the central 1 mm diameter ring around the fovea. The SFCT was defined as a perpendicular distance between the Bruch’s membrane and the choroido-scleral junction measured at the center of the fovea.

The OCTA (SD-OCT, Nidek; Model RS 3000 Advance 2; Fremont, CA, USA) 3 × 3 mm scan, centered at the fovea, was used to study FAZ, and VD in the superficial capillary plexus (SCP), deep capillary plexus (DCP), outer retina (OR), outer retinal chorio-capillaries (ORCC), chorio-capillaries (CC), and choroid (C). We bracketed different retino-choroidal layers for measuring VD. The SCP extended between the ILM and the inner plexiform layer/inner nuclear layer (IPL/INL) (i.e., between the ILM and 13 microns below the IPL/INL), the DCP was examined between the IPL/INL and the outer plexiform layer/outer nuclear layer (OPL/ONL) (i.e., at depth starting at 8 microns below the IPL/INL till 13 microns below the OPL/ONL), and the OR was bracketed between the OPL/ONL and the retinal pigment epithelium/Bruch’s membrane (RPE/BM) (i.e., at depth starting at 8 microns below the OPL/ONL till 71 microns above the RPE/BM). The ORCC was extended from the OPL/ONL and the RPE/BM (i.e., at depth starting at 8 microns below the OPL/ONL till 21 microns below the RPE/BM). The chorio-capillaries were bracketed at the level of the RPE/BM (i.e., at depth starting at 4 microns below the RPE/BM till 32 microns below the RPE/BM). The choroid was bracketed at the level of the RPE/BM (i.e., at depth starting at 25 microns below the RPE/BM till 63 microns below the RPE/BM). We did quantitative measurements of the retino-choroidal capillary microvascular layers using en-face projection images. We used the image quality “Auto All” function to remove projection and motion artifacts for optimizing image quality. All images were taken at a signal strength index of 90% and signal quality index of 4/5 at least three times, and the results were averaged.

The NVD, NVE, and IRMA were identified through clinical examination with direct and indirect ophthalmoscopy and 90 diopters fundoscopy lens examination. At least two types of multimodal imaging including OCT, color fundus photography, red-free fundus photography, and fluorescein angiography (FA) (Zeiss FF450 Plus IR Fundus Camera, Carl Zeiss Meditec AG, Jena, Germany) were used to confirm findings. The OCTA 3 × 3 mm scans were used to study NVn located at or near the posterior pole by centering OCTA images on the area of abnormal blood vessels to capture the entire neovascular complex (NVC). The NVD was identified as hyper-reflective tissue located at the disc or within one disc diameter from its margins, and the NVE was defined as new vessels located outside this area [7,10-12]. Two independent investigators evaluated the NVCs. They were verified by blood flow registration on corresponding B-scans with flow overlay of the vitreoretinal interface (VRI) showing extraretinal proliferation. On B-scans, NVE appeared as medium to highly reflective tissue that breached the ILM, while NVD showed highly reflective tissue protruding from the disc in a sea fan configuration. On OCTA, the NV were identified from enhanced flow signals, while areas of retinal poor perfusion/non-perfusion showed minimal or absent retinal flow signal. IRMA did not show signs of leakage on FFA, and on OCT scans, these were identified as vascular abnormalities that had not breached the ILM [13]. Vessel abnormalities away from the posterior pole were detected with color fundus photography, red-free fundus photography, and fluorescein angiography.

The primary outcome parameter was OCTA features in the subjects having NVn in PDR.

Sample size calculation

From the previous record, the expected prevalence of NVE in PDR is 66% [5]. Taking a 95% confidence level and 80% power of the study, the sample size came to be 50 eyes. However, we studied 79 eyes affected with PDR.

Statistical analysis

Statistical analysis was performed using Statistical Package for the Social Sciences (SPSS) version 24.0 (IBM SPSS Statistics, Armonk, NY, USA). Demographic, clinical, and imaging data were analyzed quantitatively. Quantitative data were non-parametric and expressed in median values with an interquartile range. The association between NVn and OCTA parameters was found by comparing mean values of OCTA parameters between classified groups (NVD/NVE/IRMA/VH present or absent) using the Mann-Whitney U test in the case of two groups and the Kruskal-Wallis H test in the case of more than two groups. The p-values were calculated to find the statistical significance of the association, and p<0.05 was considered statistically significant.

## Results

In the present study, 79 eyes of 41 subjects, including 28 (68%) males and 13 (32%) females, had PDR and met our inclusion criteria for evaluating the association of NVn with OCTA parameters. The mean age of our subjects was 54.22±7.1 (range: 40-70) years. The mean duration of diabetes was 15.51±3.61 (range: 10-23) years. The mean fasting blood sugar was 160.07±66.72 (range: 78-284) mg/dL, the mean postprandial blood sugar was 235.04±92.71 (range: 110-386) mg/dL, and the mean HbA1C was 9±1.95 (range: 7%-12.4%). Among study subjects, 32 (78%) patients also had hypertension as a comorbidity; the mean systolic blood pressure was 157.3±22.47 (range: 115-204) mmHg, and the mean diastolic blood pressure was 93.7±15.17 (range: 74-132) mmHg. Of 41 subjects, 18 (44%) had a normal lipid profile and 23 (56%) had an abnormal lipid profile.

There were 79 affected eyes with vascular abnormalities such as NVD, NVE, IRMA and VH.

All subjects complained of diminution of distance and near vision, metamorphopsia, decreased CS, and defective CV. The distance mean BCVA ranged from LogMAR 0.6 (20/80; 6/24) to LogMAR 1.0 (20/200; 6/60), and the mean BCVA was LogMAR 0.88±0.32 (20/125; 6/38). The mean refractive error was 0.41±0.95 diopters. The mean IOP was 17.3±4.27 mmHg. On Pelli Robson Charts, 69/79 eyes had CS less than 1.5. The mean CS was 0.97±0.37. The Farnsworth D-15 test showed that 40/79 eyes had a defective CV ranging from mild to severe tritanopia. The Amsler grid examination showed that 73/79 eyes had metamorphopsia.

The subjects presented with OCT features such as posterior vitreous detachment (10/79), vitreo-macular adhesion (30/79), thick hyaloid (25/79), spongy macular edema (65/79), hyperreflective foci (10/79), hard exudates (50/79), neurosensory detachment (60/79), epiretinal membrane (5/79), and inner segment-outer segment disruption (55/79).

The OCTA showed an enlarged and distorted FAZ and macular ischemia with disintegrated vascular arcades. We identified the NVD, NVE, and IRMA at or near the posterior pole in 3 × 3 mm scans centered on the fovea and the region around it. The NVE and IRMA not located within this area were identified with other forms of multimodal imaging. The FAZ and VD parameters were evaluated from 3 × 3 mm scans centered on the fovea. Table 1 shows the number of eyes having NVD, NVE, IRMA, and VH.

**Table 1:** Presentation of eyes having proliferative diabetic retinopathy NVD: neovascularization disc, NVE: neovascularization elsewhere, DA: disc area, IRMA: intra-retinal microvascular abnormalities, VH: vitreous hemorrhage

Serial number	Presentation	Number of eyes n=79 (100%)
1	NVD	
	NVD absent	60 (76%)
	NVD < 1/3 DA present	9 (11%)
	NVD > 1/3 DA present	10 (13%)
2	NVE	
	NVE absent	15 (19%)
	NVE < ½ DA present	40 (51%)
	NVE > ½ DA present	24 (30%)
3	IRMA	35 (44%)
4	VH	6 (7%)

Out of 79 eyes, 60 (76%) eyes did not have NVD, nine (11%) eyes had NVD < 1/3 DA, 10 (13%) eyes had NVD > 1/3 DA, 15 (19%) eyes did not have NVE, 40 (51%) eyes had NVE < ½ DA, 24 (30%) eyes had NVE > ½ DA, 35 (44%) eyes had IRMA, and six (8%) eyes had mild VH. Of 79 eyes, 15 (19%) eyes exclusively had NVD only, 64 (81%) eyes exclusively had NVE only, and four (5%) eyes had both NVD and NVE. Table 2 shows an association between NVD with retino-choroidal OCT and OCTA parameters.

**Table 2:** Association between neovascularization disc and optical coherence tomography angiography parameters *NVD present includes NVD < 1/3 DA and NVD > 1/3 DA **Statistically significant OCT: optical coherence tomography, OCTA: optical coherence tomography angiography, NVD: neovascularization disc, SFCT: sub-foveal choroidal thickness, CFT: central foveal thickness, FAZ: foveal avascular zone, SCP: superficial capillary plexus, DCP: deep capillary plexus, OR: outer retina, ORCC: outer retinal chorio-capillaries, CC: chorio-capillaries

Serial number	OCT and OCTA parameter mean and median values	No NVD (n=60)	NVD present^* ^(n=19)	p-value
1	SFCT (microns)	265.19±55.27; 276 (209-301)	307.6±29.72; 299 (289.5-330)	0.08
2	CFT (microns)	314.47±103.20; 282 (250.8-352)	341.40±153.23; 312 (227-470.5)	0.83
3	FAZ area (mm^2^)	0.38±0.29; 0.31 (0.24-0.42)	0.59±0.24; 0.56 (0.40-0.79)	0.01**
4	FAZ perimeter (mm)	2.96±1.67; 2.73 (1.74-3.55)	2.34±1.68; 1.46 (1.43-3.68)	0.21
5	FAZ circularity	0.36±0.11; 0.33 (0.29-0.44)	0.44±0.11; 0.44 (0.35-0.52)	0.13
6	SCP (foveal)	5.84±8.94; 3.0 (0.0-6.2)	0.0±0.0; 0.0 (0.0-0.0)	0.005**
7	SCP (parafoveal)	13.11±12.28; 9.1 (5.0-14.0)	6.35±1.63; 7.0 (4.6-7.8)	0.16
8	SCP (perifoveal)	17.60±14.20; 15.0 (7.0-19.2)	13.35±2.70; 12.5 (11.6-15.5)	0.57
9	SCP whole	12.66±11.47; 9.50 (5.0-15.2)	7.0±2.34; 6.0 (5.5-9.0)	0.23
10	DCP (foveal)	3.23±8.72; 0.0 (0.0-2.0)	0.60±0.89; 0.0 (0.0-1.5)	0.85
11	DCP (parafoveal)	6.49±8.63; 3.0 (1.0-9.0)	2.90±4.12; 1.0 (0.2-6.5)	0.26
12	DCP (perifoveal)	10.72±10.59; 7.5 (3.0-15.0)	7.95±5.79; 8.0 (2.6-13.2)	0.86
13	DCP whole	6.93±8.80; 4.5 (2.0-8.0)	4.40±3.43; 3.0 (1.5-8.0)	0.75
14	OR (foveal)	6.18±7.48; 4.0 (1.0-9.2)	3.0±2.34; 2.0 (1.5-5.0)	0.64
15	OR (parafoveal)	6.05±7.16; 3.0 (1.0-10.2)	2.65±4.17; 0.8 (0.2-6.0)	0.19
16	OR (perifoveal)	5.16±6.15; 2.0 (0.9-8.0)	1.90±2.88; 0.8 (0.2-4.1)	0.20
17	OR whole	5.57±6.20; 3.0 (1.0-10.2)	2.60±3.05; 1.0 (1.0-5.0)	0.23
18	ORCC (foveal)	20.12±16.85; 15.5 (9.8-28.0)	8.20±3.27; 8.0 (5.5-11.0)	0.05^**^
19	ORCC (parafoveal)	20.82±14.11; 17.0 (10.8-26.0)	15.25±4.30; 16.8 (10.8-19.0)	0.62
20	ORCC (perifoveal)	21.15±13.87; 18.0 (10.8-24.0)	17.20±4.22; 19.0 (13.5-20.0)	0.95
21	ORCC whole	20.53±14.33; 16.5 (10.5-24.2)	13.80±3.56; 15.0 (10.5-16.5)	0.40
22	CC (foveal)	15.60±14.95; 11.0 (5.8-19.2)	8.80±2.04; 8.0 (7.0-11.0)	0.45
23	CC (parafoveal)	17.32±13.97; 14.0 (9.0-21.8)	14.75±7.15; 11.5 (9.6-21.5)	0.84
24	CC (perifoveal)	16.89±14.5; 13.0 (7.6-19.2)	12.5±3.64; 12.5 (9.5-15.5)	0.78
25	CC whole	16.27±13.65; 12.0 (7.8-19.2)	12.20±4.20; 11.0 (9.0-16.0)	0.73
26	Choroid (foveal)	18.76±17.60; 12.0 (9.0-18.0)	11.20±5.80; 10.0 (7.0-16.0)	0.41
27	Choroid (parafoveal)	18.89±14.36; 14.2 (10.0-22.5)	12.56±1.49; 12.0 (11.2-14.2)	0.57
28	Choroid (perifoveal)	18.42±14.55; 14.0 (10.0-19.2)	13.4±2.99; 14.0 (10.5-16.0)	0.93
29	Choroid whole	18.27±14.61; 14.0 (9.0-18.8)	12.20±2.28; 12.0 (10.0-14.5)	0.49

Both the CFT (p=0.83) and SFCT (p=0.08) were higher in NVD eyes. The presence of NVD had a significant association with a larger FAZ area (p=0.01). These eyes had lower VD in all layers, although it was significantly lower only in SCP foveal (p=0.005) and ORCC foveal (p=0.05). The NVD eyes, however, had lesser FAZ perimeter (p=0.21) and more FAZ circularity (p=0.13) than eyes without NVD. Table 3 shows an association between NVE and retino-choroidal OCT and OCTA parameters.

**Table 3:** Association between neovascularization elsewhere and optical coherence tomography angiography parameters *Statistically significant OCT: optical coherence tomography, OCTA: optical coherence tomography angiography, NVE: neovascularization elsewhere, DA: disc area, SFCT: sub-foveal choroidal thickness, CFT: central foveal thickness, FAZ: foveal avascular zone, SCP: superficial capillary plexus, DCP: deep capillary plexus, OR: outer retina, ORCC: outer retinal chorio-capillaries, CC: chorio-capillaries

Serial number	OCT and OCTA parameter mean and median values	No NVE (n=15)	NVE < ½ DA (n=40)	NVE > ½ DA (n=24)	p-value
1	SFCT (microns)	253.98±49.18; 250.5 (205.0-290.5)	290.68±58.30; 299.0 (280.0-322.0)	287.33±57.48; 304.0 (226.2-332.8)	0.01^*^
2	CFT (microns)	289.69±81.34; 274.0 (243.5-318.8)	334.26±95.94; 312.0 (264.0-400.0)	393.5±161.39; 342.0 (276.2-554.0)	0.03*
3	FAZ area (mm^2^)	0.45±0.30; 0.4 (0.3-0.4)	0.31±0.22; 0.3 (0.1-0.4)	0.29±0.28; 0.22 (0.1-0.5)	0.009*
4	FAZ perimeter (mm)	3.13±1.86; 2.8 (1.5-4.0)	2.58±1.14; 2.4 (1.7-3.3)	2.66±1.52; 2.6 (1.1-4.0)	0.67
5	FAZ circularity	0.38±0.10; 0.4 (0.3-0.4)	0.36±0.12; 0.3 (0.3-0.4)	0.31±0.10; 0.3 (0.2-0.4)	0.07
6	SCP (foveal)	5.19±9.33; 2.0 (0.0-5.0)	5.89±9.26; 2.0 (0.0-8.0)	5.92±5.64; 5.5 (0.2-8.5)	0.48
7	SCP (parafoveal)	12.57±11.74; 9.4 (6.2-13.2)	12.82±14.13; 8.0 (4.0-26.0)	12.94±10.36; 9.0 (5.5-17.8)	0.56
8	SCP (perifoveal)	16.92±13.18; 14.8 (7.2-19.0)	17.57±16.05; 15.0 (4.0-20.0)	18.63±13.50; 13.6 (11.5-17.8)	0.92
9	SCP whole	11.88±11.04; 8.5 (5.2-13.8)	12.84±13.36; 9.0 (3.0-18.0)	13.17±8.60; 10.5 (7.2-17.2)	0.59
10	DCP (foveal)	2.92±8.60; 0.0 (0.0-1.0)	4.68±10.41; 1.0 (0.0-3.0)	1.08±2.35; 0.0 (0.0-1.0)	0.26
11	DCP (parafoveal)	6.71±8.04; 6.0 (1.0-9.0)	7.05±11.17; 2.0 (1.0-7.0)	3.22±3.65; 2.0 (0.6-4.5)	0.35
12	DCP (perifoveal)	11.25±9.21; 9.8 (4.1-15.0)	11.65±14.34; 5.0 (1.8-15.5)	5.95±5.79; 3.5 (2.2-10.0)	0.11
13	DCP whole	7.0±8.19; 6.0 (2.0-8.0)	8.05±11.38; 3.0 (2.0-9.0)	3.83±3.21; 3.0 (1.2-6.2)	0.43
14	OR (foveal)	6.71±7.75; 3.0 (1.0-10.0)	6.26±7.80; 6.0 (1.0-7.0)	2.58±2.64; 2.0 (0.0-4.8)	0.22
15	OR (parafoveal)	6.75±7.01; 4.2 (1.0-11.0)	6.12±8.27; 2.0 (1.0-10.0)	1.70±2.74; 1.0 (0.1-1.8)	0.02*
16	OR (perifoveal)	5.68±6.18; 2.5 (1.0-9.3)	5.48±6.67; 2.0 (1.0-8.0)	1.25±2.16; 0.2 (0.0-1.8)	0.005*
17	OR whole	6.21±6.18; 4.0 (1.0-11.0)	5.55±6.87; 2.0 (1.0-8.0)	1.83±2.16; 1.5 (0.2-2.0)	0.02*
18	ORCC (foveal)	21.51±17.44; 14.0 (10.0-30.0)	14.84±2.75; 13.0 (4.0-22.0)	18.0±18.06; 13.5 (6.5-17.0)	0.43
19	ORCC (parafoveal)	22.03±13.35; 18.0 (11.0-26.0)	17.65±13.5; 15.0 (6.0-25.0)	18.67±15.94; 13.6 (8.5-20.0)	0.17
20	ORCC (perifoveal)	21.48±12.59; 18.5 (13.0-23.4)	20.42±15.11; 17.0 (10.0-31.0)	19.31±15.32; 15.5 (7.4-19.9)	0.41
21	ORCC whole	21.88±14.17; 17.0 (12.0-28.0)	16.47±11.77; 16.0 (8.0-22.0)	18.75±16.24; 14.5 (7.0-19.2)	0.29
22	CC (foveal)	16.09±13.70; 11.5 (6.2-20.5)	11.73±13.19; 8.0 (4.0-12.0)	16.91±19.78; 9.5 (3.5-19.2)	0.20
23	CC (parafoveal)	19.17±12.31; 16.9 (11.1-25.4)	12.11±13.27; 9.0 (1.0-19.0)	17.10±17.86; 12.0 (4.2-17.6)	0.01*
24	CC (perifoveal)	18.15±13.63; 17.0 (9.9-20.0)	12.71±13.34; 9.0 (4.0-19.0)	16.67±16.93; 12.7 (6.5-13.0)	0.09
25	CC whole	17.58±12.07; 15.0 (11.0-20.8)	11.64±12.78; 8.0 (4.0-12.0)	16.67±17.76; 12.0 (5.2-15.0)	0.01*
26	Choroid (foveal)	18.52±17.77; 11.0 (9.2-18.0)	18.05±17.21; 14.0 (6.0-21.0)	17.67±16.07; 12.0 (6.8-20.5)	0.98
27	Choroid (parafoveal)	19.28±13.78; 15.0 (11.0-22.0)	17.21±14.41; 13.0 (7.0-28.5)	17.35±15.12; 11.8 (10.0-14.8)	0.40
28	Choroid (perifoveal)	18.43±13.83; 14.2 (10.2-18.8)	17.17±14.23; 14.0 (9.0-22.0)	18.27±16.37; 12.5 (9.5-15.5)	0.70
29	Choroid whole	18.29±13.93; 14.5 (10.0-18.0)	17.26±14.72; 14.0 (8.0-24.0)	17.26±15.77; 11.5 (8.2-15.0)	0.55

Both the CFT (p=0.03) and SFCT (p=0.01) had higher values in affected eyes. Eyes without NVE had a larger FAZ area (p=0.009) and perimeter (p=0.67) than the other two categories. The circularity index was highest in the “no NVE” group (p=0.07). On comparing eyes without NVE and NVE < ½ DA, the latter had more VD in SCP and DCP while lower VD in the remaining layers. On comparing eyes without NVE, NVE < ½ DA, and NVE> ½ DA, the latest had the highest VD in SCP and lowest VD in DCP and OR. In the remaining three plexi, ORCC, CC, and choroid, eyes with NVE > ½ DA had a higher VD than NVE < ½ DA but lower than eyes with “no NVE.” The VD at OR was significantly different among the three groups (OR parafoveal: p=0.02, OR perifoveal: p=0.005, OR whole: p=0.02) with the highest VD in normal eyes and lowest in eyes having NVE > ½ DA. Table 4 shows an association between VH and IRMA, and retino-choroidal OCT and OCTA parameters.

**Table 4:** Association between vitreous hemorrhage and intra-retinal microvascular abnormalities, and optical coherence tomography and optical coherence tomography angiography parameters *Statistically significant OCT: optical coherence tomography, OCTA: optical coherence tomography angiography, VH: vitreous hemorrhage, IRMA: intra-retinal microvascular abnormality, SFCT: sub-foveal choroidal thickness, CFT: central foveal thickness, FAZ: foveal avascular zone, SCP: superficial capillary plexus, DCP: deep capillary plexus, OR: outer retina, ORCC: outer retinal chorio-capillaries, CC: chorio-capillaries

Serial number	OCT and OCTA parameter mean and median values	No VH (n=73)	VH (n=6)	p-value	No IRMA (n=44)	IRMA (n=35)	p-value
1	SFCT (microns)	265.47±55.57; 295.0 (277.5-315.8)	297.17±37.47; 276.0 (209.0-300.5)	0.14	245.23±45.53; 300.0 (276.0-327.0)	296.34±52.82; 246.0 (202.0-288.8)	<0.001^*^
2	CFT (microns)	314.70±103.70; 285.0 (225.8-438.0)	334.17±146.78; 285.0 (252.5-346.0)	0.97	294.14±60.22; 276.0 (238.0-455.0)	343.89±140.44; 285.0 (257.0-321.0)	0.63
3	FAZ size (mm^2^)	0.39±0.28; 0.4 (0.1-0.7)	0.411±0.35; 0.3 (0.3-0.4)	0.78	0.38±0.15; 0.3 (0.1-0.4)	0.41±0.39; 0.4 (0.3-0.5)	0.19
4	FAZ perimeter (mm)	2.99±1.65; 1.5 (1.2-2.6)	2.01±1.64; 2.7 (1.8-3.6)	0.03^*^	2.71±1.47; 2.8 (1.7-4.0)	3.18±1.86; 2.2 (1.6-3.4)	0.37
5	FAZ circularity	0.36±0.11; 0.4 (0.3-0.4)	0.38±0.07; 0.3 (0.3-0.4)	0.47	0.38±0.11; 0.3 (0.3-0.4)	0.34±0.09; 0.4 (0.3-0.4)	0.09
6	SCP (foveal)	5.82±9.01; 0 (0-2.2)	1.17±2.40; 3.0 (0.0-6.5)	0.05^*^	5.57±9.64; 2.0 (0.0-9.0)	5.34±7.66; 3.0 (0.0-5.0)	0.94
7	SCP (parafoveal)	13.24±12.31; 6.5 (4.2-7.6)	5.91±2.20; 9.2 (5.0-14.0)	0.07	13.35±12.93; 10.0 (5.0-14.0)	11.84±10.85; 8.9 (4.4-13.2)	0.82
8	SCP (perifoveal)	17.88±14.15; 11.6 (6.0-14.2)	10.7±5.17; 15.0 (8.9-19.5)	0.18	18.30±14.97; 15.0 (8.0-19.0)	16.11±12.24; 14.0 (7.7-20.0)	0.98
9	SCP whole	12.79±11.49; 6.5 (4.2-8.0)	6.33±2.94; 10.0 (5.5-15.5)	0.10	12.64±12.04; 11.0 (7.0-14.0)	11.89±10.19; 8.0 (5.0-14.8)	0.55
10	DCP (foveal)	3.22±8.78; 1.0 (0.0-2.2)	1.17±1.32; 0.0 (0.0-1.5)	0.60	3.14±8.95; 0.0 (0.0-3.0)	2.97±7.94; 0.0 (0.0-1.0)	0.77
11	DCP (parafoveal)	6.65±8.67; 1.5 (0.2-3.0)	1.58±1.27; 4.0 (1.0-9.0)	0.11	6.94±8.25; 2.0 (1.0-6.0)	5.40±8.72; 6.0 (1.1-9.9)	0.07
12	DCP (perifoveal)	11.01±10.59; 4.0 (1.6-8.8)	4.87±4.01; 8.0 (3.5-15.1)	0.13	11.69±9.14; 4.2 (2.0-11.0)	9.10±11.67; 10.5 (4.2-15.9)	0.02^*^
13	DCP whole	7.08±8.84; 2.5 (1.8-4.0)	3.0±2.09; 5.0 (2.0-8.0)	0.22	7.25±8.38; 3.0 (1.0-8.0)	6.17±8.90; 6.0 (2.0-8.8)	0.11
14	OR (foveal)	6.22±7.52; 2.0 (1.0-5.5)	3.0±2.44; 4.0 (1.0-9.5)	0.52	5.52±6.50; 2.0 (0.0-10.0)	6.54±8.26; 3.5 (1.0-8.5)	0.87
15	OR (parafoveal)	6.11±7.19; 0.8 (0.2-4.0)	2.37±3.79; 3.0 (1.0-10.5)	0.12	5.83±6.14; 2.0 (0.2-11.0)	5.83±8.13; 4.0 (1.2-9.8)	0.16
16	OR (perifoveal)	5.20±6.19; 1.4 (0.2-3.2)	2.04±2.55; 2.0 (0.8-8.0)	0.28	5.42±6.02; 2.0 (0.2-6.0)	4.37±6.10; 2.4 (1.0-8.0)	0.12
17	OR whole	5.64±6.22; 1.0 (1.0-3.5)	2.33±2.80; 3.0 (1.0-10.5)	0.12	5.53±5.48; 2.0 (1.0-9.0)	5.20±6.83; 4.0 (2.0-10.0)	0.14
18	ORCC (foveal)	20.25±16.92; 9.5 (6.2-11.5)	8.66±4.22; 16.0 (9.5-28.0)	0.07	20.27±16.72; 13.0 (9.0-22.0)	18.24±16.57; 14.0 (10.0-30.0)	0.51
19	ORCC (parafoveal)	21.13±14.0; 13.4 (5.5-18.5)	12.45±6.71; 17.0 (11.0-26.0)	0.13	20.27±16.72; 17.0 (8.0-26.0)	18.85±13.26; 17.4 (11.0-25.2)	0.38
20	ORCC (perifoveal)	21.35±13.84; 18.0 (10.0-19.8)	15.33±6.32; 18.0 (10.5-24.0)	0.49	22.59±14.31; 17.8 (8.0-24.0)	18.77±12.25; 18.0 (13.1-23.4)	0.24
21	ORCC whole	20.74±14.31; 14.0 (7.2-16.2)	12.33±4.80; 17.0 (11.0-24.5)	0.16	21.39±14.59; 16.0 (8.0-24.0)	18.49±13.21; 17.0 (12.0-24.5)	0.39
22	CC (foveal)	15.90±14.88; 7.5 (0.8-11.0)	6.33±4.80; 11.0 (6.0-19.5)	0.08	18.29±16.03; 9.0 (2.0-17.0)	11.25±11.56; 11.5 (7.2-21.0)	0.02^*^
23	CC (parafoveal)	17.69±13.80; 10.2 (0.8-19.0)	10.75±9.95; 14.0 (9.0-21.9)	0.20	21.11±14.28; 11.0 (3.0-18.0)	12.20±11.06; 16.9 (11.0-28.0)	0.004*
24	CC (perifoveal )	17.18±14.42; 11.8 (2.5-14.2)	9.75±6.47; 13.0 (8.0-19.5)	0.22	20.78±15.13; 9.5 (4.0-18.0)	11.37±10.72; 17.1 (10.6-20.8)	0.001*
25	CC whole	16.58±13.53; 10.5 (1.5-14.5)	9.17±7.08; 12.0 (8.0-19.5)	0.18	19.60±14.28; 11.0 (4.0-15.0)	11.51±10.44; 14.5 (10.1-24.0)	0.004*
26	Choroid (foveal)	19.11±17.52; 7.0 (2.2-13.5)	8.17±7.36; 12.0 (9.0-18.0)	0.05^*^	21.57±18.88; 10.0 (6.0-16.0)	14.14±13.96; 12.5 (10.0-21.0)	0.03*
27	Choroid (parafoveal)	19.25±14.19; 11.7 (1.8-14.0)	9.13±6.02; 14.5 (10.0-23.0)	0.09	21.35±15.08; 12.0 (7.0-17.0)	14.88±11.70; 14.7 (11.0-27.8)	0.06
28	Choroid (perifoveal)	18.78±14.42; 10.5 (3.5-16.0)	9.83±5.94; 14.0 (10.2-19.5)	0.14	21.19±15.50; 12.2 (8.0-16.0)	14.22±11.27; 14.9 (11.0-24.2)	0.01*
29	Choroid whole	18.63±14.67; 10.0 (2.5-14.2)	8.83±5.70; 14.0 (10.0-19.5)	0.06	20.77±15.40; 12.0 (7.0-16.0)	14.26±11.81; 15.0 (10.2-21.8)	0.02*

Eyes having VH had a thicker SFCT (p=0.14) and CFT (p=0.97) and a larger FAZ area (p=0.78), although the difference was not significant. Notably, unaffected eyes had a larger FAZ perimeter (p=0.03) and lesser circularity (p=0.047) than those having VH. In VH, VD in all retino-choroidal layers was lesser than those without VH, but it was statistically significant in SCP foveal (p=0.05) and choroid foveal (p=0.05).

Eyes having IRMA had a thicker SFCT (p<0.001; significant) and CFT (p=0.63) and a larger FAZ area (p=0.19) and FAZ perimeter (p=0.37) but lesser circularity (p=0.09). The VD of all retino-choroidal layers was lesser, although it reduced significantly in DCP perifoveal (p=0.02), CC whole (p=0.004), and choroid whole (p=0.02).

## Discussion

Retinal NV that grows into the vitreous through a break of the ILM is the hallmark of PDR. It predicts the risk of SVL and consequently requires sensitive detection [10,11]. Chronic hyperglycemia affects the microvessels of the retina through inflammation, hypoxia, and oxidative stress [14]. The ischemia causes NVn, but even subsequent neo-flow channels to counteract non-perfusion is an abortive effort. Thus, VD of different retino-choroidal layers is bound to remain affected [6]. OCTA can study FAZ and image all layers of the retinal vasculature, including the deep layers and choroidal plexi. As DR is a retinal vascular disease, an accurate assessment of VD in the retino-choroidal plexuses appears helpful to monitor progress and know the prognosis. Additionally, the size of the FAZ and diminishing VD are closely related to vision, thus heightening the functional significance of these parameters in DR and PDR [15-17].

Fawzi et al. [6] emphasized that in PDR, there is an alteration in the flow metrics of all the capillary layers at the macula. Vaz-Pereira et al. [5] used OCTA in 23 PDR eyes with 35 neovascular complexes (NVCs), including 34% NVD and 66% NVE for visualizing blood flow and seeing retinal non-perfusion areas. Kilani et al. [7] compared OCTA images with FA images for diagnosing and localizing NV in 42 PDR eyes and found OCTA superior in 23 eyes, equivalent in 15 eyes, and inferior in four eyes. They found OCTA to be more reliable as it evades the masking effect of dye leakage in FA and identifies the posterior hyaloid membrane, which is a proliferation route for NV. However, a small field, inability to see dynamic changes, and deficiency in segmentation algorithms, especially in eyes with a pathological interface or preretinal abnormalities, are the shortcomings of the OCTA. So, corresponding B-scans with flow registration to detect all NV on OCTA are recommended. Pichi et al. [8] studied 82 PDR eyes and found that wide-field OCTA can identify NV that is not evident in ultra-widefield color fundus photos and ultra-widefield fluorescein angiography. They concluded that OCTA represents a faster and safer alternative. Although these authors studied the NVCs in PDR qualitatively, they did not study the quantitative parameters of FAZ and VD.

The FAZ is a region surrounding the fovea devoid of retinal capillaries, and it gets enlarged and acircular from microvascular abnormalities and capillary occlusion [18,19]. Features such as breaks in the border, increased tortuosity, vessel loops, and budding of capillaries into the FAZ have been encountered with increasing severity of DR [19]. Liu et al. [19] observed a significant increase in acircularity index (AI) in diabetic subjects on the transition from mild DR to severe DR (1.18±0.07 versus 1.22±0.10; p=0.010), while the FAZ area did not show a significant increase (0.33±0.15 mm^2^ versus 0.35±0.13 mm^2^; p=0.832). These authors stated that AI might be a more sensitive imaging biomarker than the FAZ area for monitoring the progression of DR. This is because the AI is measured from a perimeter calculated/standard circular perimeter of equal area, and it becomes larger when the shape becomes less smooth or less round. Thus, AI change does not require the total disappearance of capillary; even subtle changes such as alteration in capillary distribution that precedes their total disappearance can cause its increase.

Fawzi et al. [6] found that in PDR, SCP flow remained either unaltered or showed a steal phenomenon with an increasing appearance of IRMA and dilated telangiectatic vessels in SCP along with an exacerbated ischemia at the deeper plexuses. Liu et al. [19] studied VD in SCP, DCP, and FD-300, wherein FD-300 was the VD of the entire retina in a 300 μm width around the FAZ including more layers than just SCP and DCP. On comparing subjects with no DR versus severe DR, they found that with increasing severity of DR, VD in FD-300 (48.21±3.93 versus 41.87±4.67; p<0.001), SCP (47.56±4.08 versus 38.26±5.52; p<0.001), and DCP (50.26±4.54 versus 41.86±5.93; p <0.001) decreased significantly. However, the automated preset algorithm of the AngioVue system used in this study segmented and examined only two retinal capillary plexuses, the SCP and DCP.

Nesper et al. [20] found that VD in the DCP reduced significantly from 53.76±4.63 in non-proliferative diabetic retinopathy (NPDR) to 49.73±3.66 in PDR (p<0.01). They found that VD at the DCP correlated most strongly with DR severity. The DCP is more susceptible to ischemia as it supplies the OPL, which has some of the highest oxygen demands in the retina [21]. Ashraf et al. [22] observed that changes in the middle capillary plexus (MCP) and DCP accompanied early DR, while advanced DR impacted the SCP more dramatically. These authors emphasized the importance of assessing VD changes independently in different retino-choroidal layers.

The choroidal vasculature is the primary blood supply to the highly metabolically active photoreceptors. CC dropout and ischemia have been seen in the eyes of diabetic patients with and without DR [23]. Authors have observed lower perfusion density and decreased flow in the CC of the macula in diabetes even without retinopathy, and the parameters worsen with increasing severity of DR [24-26]. Gendelman et al. [27] reported a positive association between flow deficit percentage in CC flow and DR severity. From an OCTA study, Ro-Mase et al. [28] found that an impaired CC flow in DR was associated with defective visual function.

Moir et al. [29] stated that quantitative angiography indices including FAZ and VD parameters, fractal dimension (FD), vessel tortuosity (VT), and areas of flow deficits or non-perfusion within the CC are possible biomarkers for the detection and progression of both DM and DR. However, these have been inconsistently used in the literature, on account of limitations in software packages incorporated in equipment.

In our study, eyes with NVD, VH, and IRMA had lesser VD in all the retino-choroidal layers, although a higher VD in SCP was associated with NVE > ½ DA. Thus, the reduction of VD in SCP was associated with the appearance of NVD (p=0.005), but not NVE (p=0.59). As SCP VD had the highest values in NVE > ½ DA; thus, we agree with Fawzi et al. [6] that SCP shows a steal phenomenon [6]. On comparing eyes without NVE, NVE < ½ DA, and NVE> ½ DA, the latest had the highest VD in SCP and lowest VD in DCP and OR. Thus, VD in DCP and OR appeared as the most reliable parameters to determine the severity of NVE. In ORCC, CC, and choroid layers, the eyes with NVE > ½ DA had higher VD than eyes with NVE < ½ DA but lesser than the VD of unaffected eyes. It appears that an increase in the VD of the choroid is a compensatory response in eyes with a severer disease, i.e., choroidal supply may be increased in an effort to enhance the blood supply of adversely affected eyes. Thus, the VD parameters at these layers may point toward compensation of ischemia and per se not ischemia itself. The CFT and SFCT were thicker in eyes with NVD, NVE, VH, and IRMA. A larger FAZ area was associated with the appearance of NVD, VH, and IRMA, but not with NVE; a larger FAZ perimeter was associated with IRMA, but not with NVD, NVE, and VH; and a worse circularity was associated with IRMA and NVE, but not with NVD and VH. Except for acircularity, both FAZ parameters including area and perimeter were not associated with NVE, thereby indicating that acircularity is more sensitive for indicating NVE. This agrees with Liu et al. [19] who stated that circularity is a more sensitive index. IRMA was associated with a larger FAZ area, larger FAZ perimeter, and lesser circularity, indicating that the presence of IRMA indicates central ischemia.

The strength of this study was that we identified NVn and IRMA from clinical examination and multimodal imaging techniques. The shortcomings included eliminating high-risk PDR subjects with media opacities from dense VH and dense preretinal hemorrhage. For VD, we studied only a 3 × 3 mm scan at the posterior pole, and there is a possibility that VD may be variable in areas surrounding a 3 × 3 mm scan. However, smaller scans can show changes at deep capillary levels and with high resolution, while large-sized scans do not provide adequate pixel resolution to demarcate and visualize the deeper plexi [6].

## Conclusions

The association between flow metrics of different capillary layers and types of vascular abnormalities including NVn, IRMA, and VH have rarely been studied. The present study found that an increased CFT and SFCT are related to the appearance of NVD, NVE, VH, and IRMA. A larger FAZ area was associated with NVD, VH, and IRMA, but not with NVE. Eyes with NVD, VH, and IRMA had lesser VD in all the retino-choroidal layers. On comparing subjects without NVE, NVE < ½ DA, and NVE > ½ DA, the latest had the highest VD in SCP and lowest VD in DCP and OR. IRMA was associated with a larger FAZ area, larger FAZ perimeter, and lesser circularity, showing that the presence of IRMA indicates central ischemia. As FAZ parameters and VD in different layers of the retina and choroid had a significant association with the appearance of NVn, the quantitative assessment of these indices can help us monitor disease progression and predict the occurrence of further complications.
